# Giant Tunable Circular Dichroism of Large-Area Extrinsic Chiral Metal Nanocrescent Arrays

**DOI:** 10.1186/s11671-019-3220-7

**Published:** 2019-12-21

**Authors:** Liyuan Cao, Jiwei Qi, Qiang Wu, Zhixuan Li, Ride Wang, Junan Chen, Yao Lu, Wenjuan Zhao, Jianghong Yao, Xuanyi Yu, Qian Sun, Jingjun Xu

**Affiliations:** 1 The Key Laboratory of Weak-Light Nonlinear Photonics, Ministry of Education, TEDA Institute of Applied Physics and School of Physics, Nankai University, Tianjin, 300457 China; 2 Collaborative Innovation Center of Extreme Optics, Shanxi University, Taiyuan, 030006 Shanxi China

**Keywords:** Metal nanocrescent arrays, Extrinsic chirality, Circular dichroism, Lattice surface modes

## Abstract

Circular dichroism (CD) is an interesting phenomenon originating from the interaction of light with chiral molecules or other nanostructures lacking mirror symmetries in three-dimensional (3D) or two-dimensional (2D) space. While the observable effects of optical chirality are very weak in most of the natural materials, they can be designed and significantly enhanced in synthetic chiral structures, where the spatial symmetry of their component are broken on a nanoscale. Therefore, fabrication of composites capable of cheap, time-saving, and giant CD is desirable for the advanced optical technologies. Here, the giant CD of large-area metal nanocrescent array structures was investigated theoretically and experimentally. The largest value of the CD spectrum measured was larger than 0.5, and the CD spectrum was tuned effectively and extensively while maintaining a large peak intensity, which can be attributed to the selective excitation of the lattice surface modes (LSMs) by circularly polarized light. The analysis of the extrinsic chiral structure shows potential applications in chiral molecule sensing and polarizing imaging.

## Introduction

An object is chiral if its structure differs from its mirror image enantiomer [1]. Chirality is a ubiquitous property possessed by a large variety of compounds, such as biological and chemical substances, or artificial metamaterials [2]. This structural property is widely used in many fields, such as physics, biology, chemistry, and medicine [3–6]. Chiral noble metal nanostructures have been extensively studied in the last several decades, due to their tunable optical properties, which include optical rotation, asymmetric transmission of circularly polarized light, and extraordinary circular dichroism (CD) [7–14]. These exceptional properties are attributed to the strong optical response of these structures and it is generated by localized surface plasmon resonances (LSPRs). This phenomenon is sensitive to the shape, size, and surroundings of the metal nanostructures [15–17]. For these reasons, chiral metal nanostructures can be used in many applications, such as negative refraction [18–20], in the manipulation of the polarization of a light source [21–23], and in chiral molecule sensing [24, 25].

Optical chirality can also be generated in achiral metamaterials by breaking the mirror symmetry of the experimental arrangement via oblique illumination. This phenomenon is known as “extrinsic chirality” due to the lack of the twofold rotation symmetry of the compound. Extrinsic chirality was initially introduced and proved by Bunn in 1945. Zheludev and co-workers discovered the extrinsic chiral response induced by extrinsic chirality in a metal split ring. Moreover, they studied the interaction mechanism between the electric dipole and the magnetic dipole of these structures [26, 27]. Recently, Leon et al. [28] demonstrated the large circular dichroism in a metasurface composed of metal split ring arrays experimentally and theoretically. When compared to chiral metal nanostructures, extrinsic chiral metal nanostructures with a large surface are easier to be fabricated [29–34]. Furthermore, they show even stronger chiral optical properties, such as CD, which implies that the compound presents different transmissions when it interacts with left circularly polarized (LCP) or right circularly polarized (RCP) incident waves [35, 36]. In a previous work of this same research group, a large-area randomly distributed metallic crescent nanostructure was fabricated and it was proved to possess a large optical chirality [37]. However, due to the low density of the randomly distributed nanocrescents, the CD coefficient obtained in the experiment was lower than the expected one. Furthermore, the uniformity of the randomly distributed metallic crescent nanostructures presented several imperfections which prevented the use of this compound in applications. Since the array structure provides a large cell density and a good uniformity. The development of simple, well-known, and low-cost fabrication methods to produce large-area, uniform, extrinsic chiral metal array structures constitutes a new challenge to promote the use of metal chirality in applications.

In this work, periodic array of metallic crescent nanostructures with lattice constants in the range 500–1000 nm was fabricated using polystyrene (PS) microsphere array as template. The maximum value of the CD (0.51) was measured at 1270 nm for a lattice constant of 800 nm. Simulations of the proposed structure were implemented and found to be in excellent agreement with the experimental measurements. According to the simulations, the main mechanism at the basis of this intense CD effect is the selective excitation of the LSMs via the circularly polarized light. Furthermore, the tunability of the CD effect was verified experimentally by changing the lattice constant of the structure. Since the PS microspheres are commercially available, the extrinsic chirality of the periodic array of metallic crescent nanostructures can be modulated over a wide spectral range, which spans from the visible to the infrared region. The proposed sample has advantages of high dichroism, easy fabrication and standard fabrication technique compatibility, which could lead to imaging and sensing applications of circularly polarized light.

## Methods

**Fig. 1:**
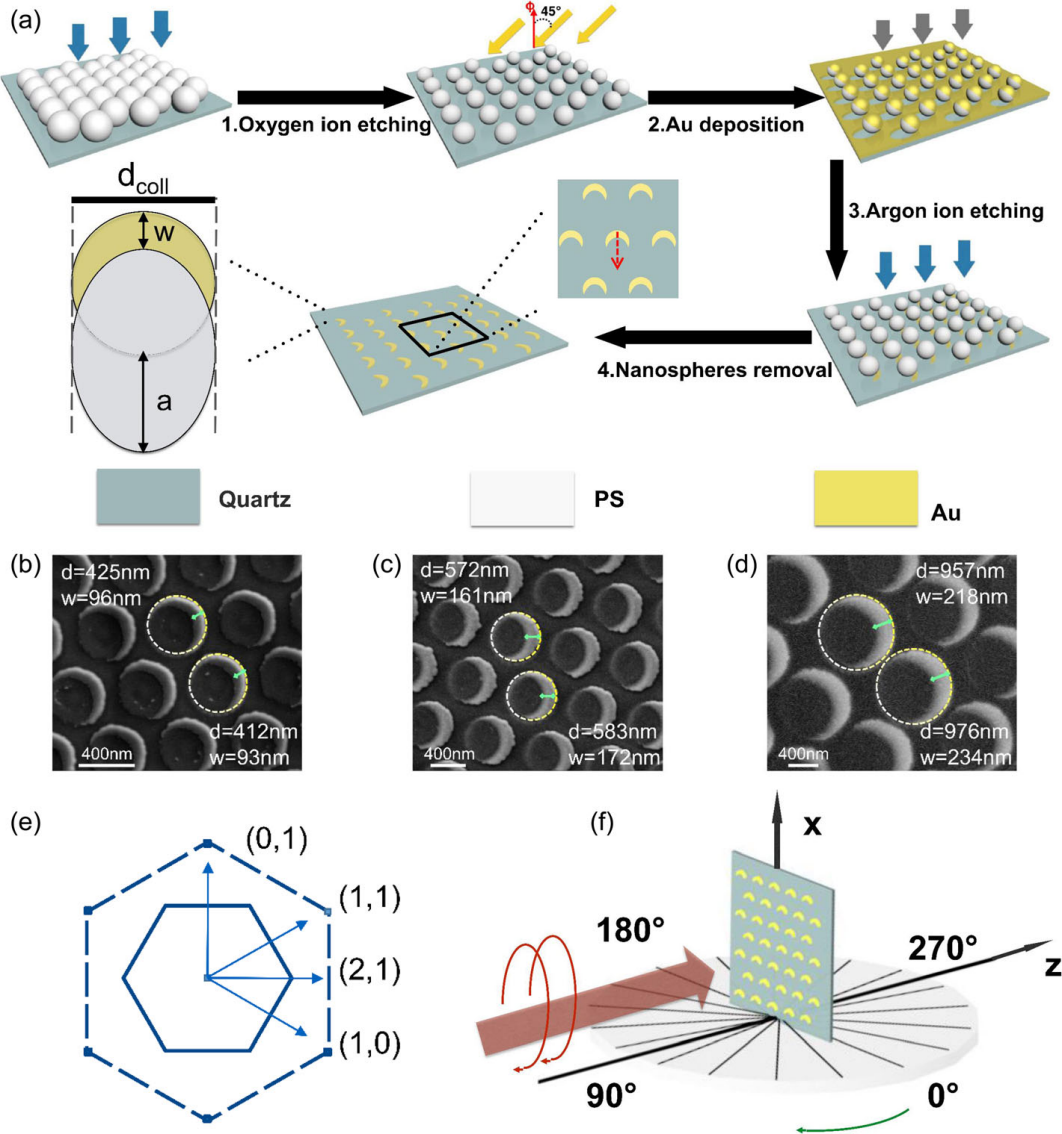
**a** Sketch of crescent preparation process and the particle geometry indicating the diameter *d*_coll_ of masking colloid and the maximum crescent width *w* of the metal. The red arrow indicates positive direction of the mirror axis of nanocrescents; **b**–**d** SEM images of the structure with lattice constants: **b** 500 nm, **c** 800 nm, **d** 1000 nm. The length of the green arrow represents the maximum width of the crescent-shaped structure *w*. The dotted line represents the diameter of the crescent structure after fitting with a circle (etched PS nanospheres); **e** The reciprocal lattice is spanned by the basis vectors (1, 0) and (0, 1). The reciprocal vector (1, 1) and (2, 1) are shown. The continuous and dashed lines in the diagram of the reciprocal lattice represent the boundaries of the first and the second Brillouin zones, respectively. **f** Schematic design of the experiment

The large-area, equilateral triangular lattice arrays of metallic crescent nanostructures with different lattice constants were fabricated using PS nanospheres of different sizes as the templates. The diameters of the PS nanospheres used in this work are 500, 650, 800, 850, and 1000 nm. The fabrication process is shown in Fig. 1a. Initially, a monolayer hexagonal close-packed array of PS spheres was formed on a pre-treated quartz surface via the self-assembly process [38]. The close-packed colloidal monolayer was then etched by forming an argon plasma for 6 min (PDC-32G-2) to obtain a non-close-packed template [39, 40]. The sample was maintained at a pressure of 0.2 mbar and the input power of the light was set to 100 mW. Successively, a 50-nm thin gold layer was deposited by ion beam sputtering coating with a tilt angle of 45°. The gold film was vertically etched by the ion beam. Finally, the nanosphere template was removed using acetone, and the large-area, equilateral triangular lattice arrays of metallic crescent nanostructures were formed. Following basic geometrical considerations, the crescent diameter can be adjusted by choosing a different diameter of the PS nanospheres. Furthermore, the film thickness is directly accessible by controlling the amount of gold deposited on the sample, and the maximum crescent width *w* of the metal is given as

$$ w=\frac{d_{coll}}{2}\left[1-\frac{\left(1-\sin \phi \right)}{\cos \phi}\right]. $$

Substituting *Φ* = 45°, used throughout the studies discussed here, in the above equation yields

$$ w=0.59\cdot \frac{d_{coll}}{2}. $$

**Fig. 2:**
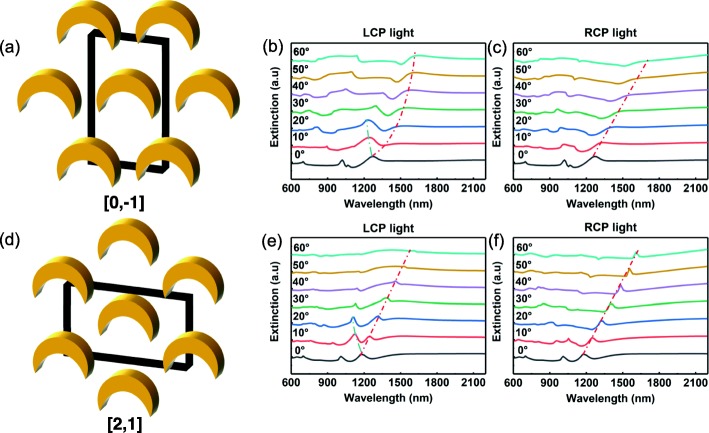
**a**, **d** Schematic of the lattice where the mirror axis of nanocrescents is oriented towards the [0, − 1] and [2, 1] crystal axis, respectively (the blocks represent the crystal cell); **b**, **c** extinction spectrums recorded by employing an incident light with left and right circular polarization, respectively. The mirror axis of nanocrescents is directed towards the [0, − 1] direction of the crystal lattice; **e**, **f** extinction spectrums recorded by employing an incident light with left and right circular polarization, respectively. The mirror axis of nanocrescents is directed towards the [2, 1] direction of the crystal lattice

It must be noted that in reality, deviations from the idealized geometry suggested in Fig. 1a occur. The *w*, seen in the scanning electron images, Fig. 2b–d, is slightly smaller than the ideal case. As additional systematic uncertainties, etching and aggregation of colloids should be taken into account. The red arrow in Fig. 1a defines the positive direction of the mirror axis of nanocrescents which is towards the opening direction of nanocrescents. As shown in Fig. 1b–d, the direction of mirror axis of nanocrescents is consistent, and this could be determined via the tilting deposit process and was controlled artificially. The metal nanocrescents regularly arrange within a relatively large area. However, the orientation of the lattice is difficult to control outside the optical measurement area, which measures a few square millimeters, due to fabrication flaws. Therefore, the relative orientation between the direction of metal nanocrescents and the equilateral triangular lattice is random.

The sample with extrinsic chiral response was modeled using a Maxwell’s equation solver, which is based on the Finite-Difference Time-Domain (FDTD) method. The metal nanocrescent array structure with lattice constant of 800 nm (i.e., the sample formed with PS microspheres of 800 nm diameter) was selected to carry out the simulation. While in the experiment the relative orientation between the mirror axis of nanocrescents and the lattice is random, in the simulation, the mirror axis of nanocrescents was chosen towards the [0, − 1] and [2, 1] crystal axis, as shown in Fig. 2a, d, for the sake of simplicity. Here, the quartz constitutes the substrate and the Au is the metal, as in the Johnson and Christy’s model. Periodic boundary conditions were applied along the *x*- and *y*-directions. The FDTD mesh size was set to 2 nm to provide an accurate calculation of the plasmonic effect. By directing left circularly and right circularly polarized light onto the sample and by simultaneously rotating the sample around the rotational symmetry axis of the metal nanocrescent, the incident angle can be changed, as shown in Fig. 1e.

## Results and Discussion

The surface-lattice resonances (SLRs) in a two-dimensional array of nanoparticles have been widely studied. The extinction features in the spectrum result from the lattice surface modes (LSMs), which are generated by grazing diffraction orders or Rayleigh anomalies (RAs) [28, 41]. In this work, the size of the nanoparticles is approximated by the lattice constant. For this reason, the extinction features present in the spectrum, which result from the LSMs, can still be observed although the environmental refractive index asymmetry was introduced by adding the quartz substrate [42]. Moreover, a significant red-shift that originated from the Rayleigh anomaly condition, due to the strong coupling of the LSPR with the neighboring metal particles, can be observed [43]. These phenomena are presented in detail in the following sections of this work.

When the mirror axis of nanocrescents points towards the [0, − 1] lattice crystal axis and the incident angle of the light is 0°, the extinction spectra generated by the two circularly polarized light overlap. Furthermore, each spectrum exhibits three extinction peaks, which are located at 697 nm, 1019 nm, and 1265 nm, respectively (Fig. 2b, c). Due to the low intensity of the extinction peak located at 697 nm and its little contribution to the CD effect, no further study on this feature was performed. The extinction peak located at 1265 nm is mainly induced by the LSMs (± 1, 0), (1, 1), and (− 1, − 1), whereas the feature appearing at 1019 nm is mostly generated by the LSMs (2, 1) and (− 2, − 1). The introduction of the crescent structure removes the degeneracy since it is characterized by a relatively low degree of symmetry. As a result, the extinction peak at 1019 nm splits when it is illuminated by a light with a 0° angle of incidence. When the incidence angle *θ* increases and the mirror axis is parallel to the [0, − 1] direction of the crystal lattice, the resonance equation of the LSMs can be written as follows:

${\lambda}_{RA}^{\pm}\left(\theta \right)=\frac{\sqrt{3}}{2}\varLambda n\left[1\pm \frac{\sqrt{3}}{2}\sin \left(\theta \right)\right]$ for the (±1, 0), (1, 1), and (−1, −1) modes;

${\lambda}_{RA}^{\pm}\left(\theta \right)=\frac{1}{2}\varLambda n\left[1\pm \sin \left(\theta \right)\right]$ for the (2, 1), (−2, −1) modes. where *Λ* is the lattice constant of the equilateral triangular lattice, which measures 800 nm; the symbol ± (positive or negative) depends on the first digit of the LSMs; *n* is the effective refractive index of the surrounding of the equilateral triangular lattice, which assumes almost identical values (1.25) for every LSM. By introducing these values in the expressions above, the extinction peak induced by the LSMs (± 1, 0), (1, 1), and (− 1, − 1) should appear at 866 nm, whereas the feature induced by the LSMs (2, 1) and (− 2, − 1) at 500 nm. However, the simulation results show that these peaks are located at 1265 nm and 1019 nm, which means that they are largely red-shifted from the calculated ones. The red-shift is caused by the strong coupling of the LSPR modes with the neighboring metal nanoparticles [43]. The coupling strength of the LSPR modes with the neighboring metal nanocrescents is different for different geometric configurations and modes and this induces a red-shift in their optical response. In this work, the different values of the red-shifts are dependent on the effective refractive index, *n*, in the resonance equation. The effective refractive index measured when the mirror axis of nanocrescents is towards the [0, − 1] crystal axis and *θ* = 0° is 1.46 for the LSMs (± 1, 0), (1, 1), and (− 1, − 1), and 2.04 for the LSMs (2, 1) and (− 2, − 1). As the incident angle increases, the degeneracy is removed and the extinction peaks at 1265 nm and 1019 nm become broader or split. The removal of the degeneracy is a very complicated process since the excitation efficiencies of the LSPR modes change with different trends upon the increase of *θ*. Therefore, this work focuses on the main factor which gives rise to the huge CD effect. As shown in Fig. 2b, c, the extinction peak located at 1265 nm is blue-shifted upon the increase of the LCP light incident angle, but this phenomenon is not observed in the extinction spectra where RCP light is used. These results show a significant difference between the extinction spectra measured with RCP and LCP incident light and this may be related to the huge CD effect measured; while LCP light can excite the (− 1, 0) and (− 1, − 1) LSMs, this does not happen with RCP light.

When the mirror axis of nanocrescents is aligned with the [2, 1] axis of the lattice, similar results are obtained. As shown in Fig. 2e, f, when *θ* = 0°, the extinction spectra for the two types of circularly polarized incident light overlap. Moreover, each spectrum also exhibits three extinction peaks, located at 697 nm, 1019 nm, and 1171 nm, respectively. The extinction peak located at 697 nm was not taken into consideration in the following analysis. The observations suggest that the extinction peak located at 1171 nm is mainly induced by the LSMs (0, ± 1), whereas the one positioned at 1019 nm may be generated by the LSMs (− 1, 1), (1, − 1), (1, 2), and (− 1, − 2). Upon the increase of the incidence angle, *θ*, and when the mirror axis is parallel to the [2, 1] direction of the lattice, the resonance equation of the LSMs can be written as follows:

${\lambda}_{RA}^{\pm}\left(\theta \right)=\frac{\sqrt{3}}{2}\varLambda n\left[1\pm \sin \left(\theta \right)\right]$ for the (0, ±1) modes;

${\lambda}_{RA}^{\pm}\left(\theta \right)=\frac{1}{2}\varLambda n\left[1\pm \frac{\sqrt{3}}{2}\sin \left(\theta \right)\right]$ for the (−1, 1), (1, −1), (1, 2), and (−1, −2) modes.

The sign ± (positive or negative) depends on the second digit of the LSMs. When *θ* = 0°*, n* is 1.35 for the LSMs (0, ± 1), whereas it measures 2.04 for the LSMs (− 1, 1), (1, − 1), (1, 2), and (− 1, − 2). When the incident angle increases, the extinction peaks at 1171 nm and 1019 nm become broader or split. Similarly, when the mirror axis of nanocrescents is parallel to the [0, − 1] crystal axis, the most significant difference between the extinction spectra recorded using RCP and LCP incident light is a series of extinction peaks. They are blue-shifted compared to the peak located at 1171 nm. Moreover, upon the increase of the incident angle, they only appear in the extinction spectra measured via the LCP incident light, but that cannot be observed if RCP light is used. This observation may explain the measured huge CD effect since only the LCP light can excite the (0, − 1) LSM. The selective excitation of the LSMs via left and right circularly polarized light may then be responsible for the huge CD effect observed in extrinsic chiral array structures and this observation is consistent with Ref. [28].

**Fig. 3:**
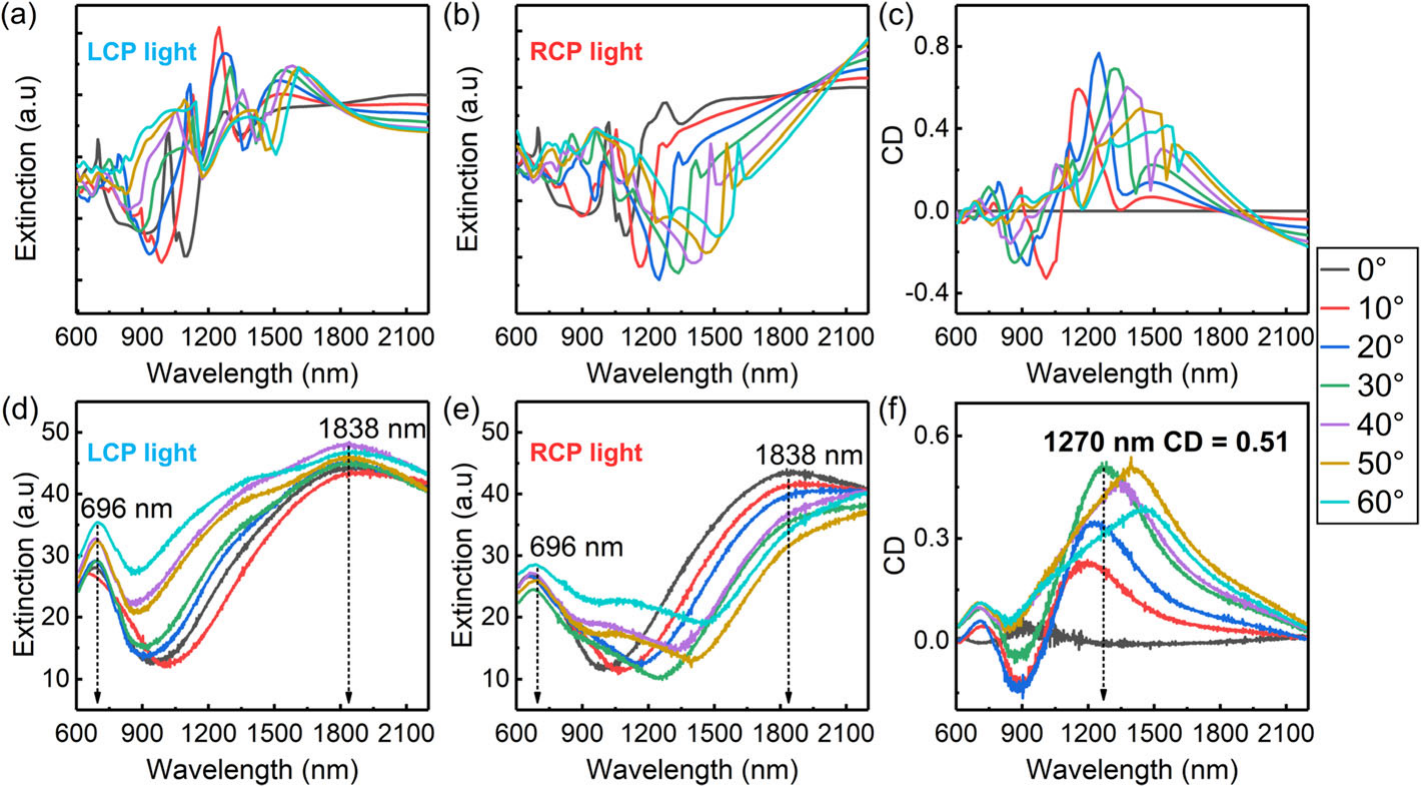
Simulated and measured extinction and CD spectra. **a**–**c** Simulated additive extinction spectra for different incidence angles of the circularly polarized light: **a** LCP, **b** RCP, and the CD spectra. **d**–**f** Measured extinction spectra for different incidence angles of the circularly polarized light: **d** LCP, **e** RCP and the CD spectra

An experimental measurement was performed to obtain the extinction spectra and the CD spectra of the samples. A measurement system, which constitutes an ultraviolet–visible–near-infrared spectrophotometer, was developed. The light is driven through a Glan–Taylor prism and a broad-spectrum quarter-wave plate to ensure that circular polarization can be achieved and that the sample is irradiated under a certain angle. This angle can be precisely controlled by rotating the sample stage. The CD coefficient can be calculated using the following equation:

$$ CD=\frac{L_{ext}-{R}_{ext}}{L_{ext}+{R}_{ext}}, $$

where *L*_*ext*_ and *R*_*ext*_ are the extinction intensities of the metal nanocrescents measured with the spectrophotometer via LCP light and RCP light, respectively. The results are shown in Fig. 3d, e, whereas the CD spectra are presented in Fig. 3f. To approximate the simulations to the experimental conditions, the extinction spectra of the two configurations were superimposed (Fig. 3a, b) and the simulated CD coefficients were calculated (Fig. 3c). The simulations are in good agreement with the experimental results, especially in the case of the CD spectra. As shown in Fig. 3d, e, when *θ* = 0°, the extinction spectra measured by LCP and RCP incident light are almost identical. Furthermore, two prominent extinction peaks located at 696 nm and 1838 nm are present. The results suggest that the extinction peak at 696 nm is generated by the high-order LSPR mode. The extinction peak at 1838 nm may instead arise due to the LSMs (± 1, 0), (1, 1), (− 1,− 1), (0, ± 1) and the LSPR dipole mode. Upon the increase of *θ*, the extinction peak at 696 nm initially decreases and then it increases again, although its intensity is different in the LCP and RCP spectra. This observation is consistent with the conclusions of the previous work of this research group. The extinction peak at 1838 nm presents only a slight change and a new extinction peak located at 1390 nm arises upon the increase of *θ* and when LCP incident light is used. This results in the excitation of the (− 1, 0), (− 1, − 1), and (0, − 1) LSMs. When the sample is excited via RCP incident light, the extinction peak at 1838 nm red-shifts and its intensity becomes weaker as *θ* increases. Although there are no extinction peaks located at 1390 nm, a new feature appears at 1080 nm when *θ* is increased and this may be generated by the LSPR modes. As shown in Fig. 3f, upon an increase in *θ*, a major CD peak arises and red-shifts. When *θ* = 30°, the maximum value of the CD coefficient (0.51) can be measured at 1270 nm. The selective excitation of the (− 1, 0), (− 1, − 1), and (0, − 1) LSMs via the circularly polarized light triggers the mechanism responsible for the huge CD effect. Due to the flaws in the production process, the extinction and CD peaks obtained in the experiment are slightly broader when compared to the simulated ones.

**Fig. 4:**
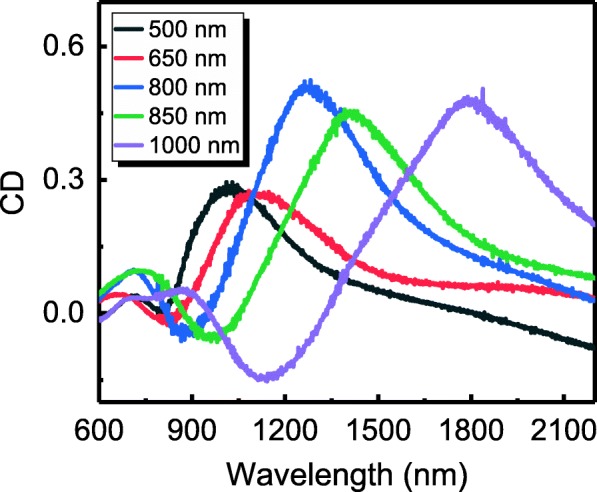
CD spectra of metal nanocrescent arrays with diameter in the range of 500–1000 nm; the incident angle of the light source is 30°

In addition, this work shows that the extrinsic chirality of the array of metallic crescent nanostructures can be tuned by adjusting the diameter of the PS microsphere. Figure 4 shows the CD spectra of several metal nanocrescent arrays with different lattice constants (i.e., diameter of the PS nanospheres) in the range of 500–1000 nm with *θ* = 30°. Upon the increase of the lattice constant, the peak of the CD spectra red-shifts from 1019 to 1799 nm, and the CD coefficient remains relatively large (> 0.25). Since PS microspheres with diameters between 50 nm and 10 μm are available commercially, the extrinsic chirality of the structure can be modulated for its application over a wide range of wavelengths ranging from visible to infrared.

## Conclusions

In summary, we have demonstrated that the large-area metal nanocrescent array structures show a considerable extrinsic chiral effect, as well as a high modularity and a simple fabrication method. Samples with different lattice parameters were successfully fabricated and the CD effect was studied theoretically and experimentally. The largest CD coefficient (> 0.5) was measured at 1270 nm using an angle of incidence of 30° in a metal crescent array with a 800-nm period. Furthermore, the CD spectrum of such structures can be extensively tuned, while maintaining a large peak intensity, by changing the diameter of the PS microspheres. The locations of the CD peaks vary from 1019 to 1799 nm, upon a change in the lattice constant in the range of 500–1000 nm. The simulations are in good agreement with the experimental results and the large and tunable extrinsic chiral effect of the samples can be attributed to the selective excitation of the LSMs induced by LCP and RCP. The demonstrated structure could be useful in remote sensing and polarization imaging.

## Data Availability

All data generated or analyzed during this study are included in this published article.

## Abbreviations

LCP: left circularly polarized
RCP: right circularly polarized
CD: circular dichroism
LSPRs: localized surface plasmon resonances
LSMS: lattice surface modes
SLRS: surface-lattice resonances
SEM: scanning electron microscope
